# Can routinely collected electronic medical record (EMR) data support hospital resource allocation? A retrospective analysis of 332,711 presentations to a public quaternary teaching hospital in South Australia (2020–2025)

**DOI:** 10.1186/s12913-026-14448-8

**Published:** 2026-03-27

**Authors:** Madison Bills, Taryn Bessen, Matthew Williams, Annie Conway, Brandon Stretton, Tony Loan, Joshua Kovoor, Danny Liew, Stephen Bacchi, Gerry O’Callaghan

**Affiliations:** 1 Adelaide EpiCentre, Central Adelaide Local Health Network, SA Health, Adelaide, South Australia Australia; 2 Medical Services, Central Adelaide Local Health Network, SA Health, Adelaide, South Australia Australia; 3 School of Medicine, College of Health, Adelaide University, Adelaide, South Australia Australia; 4 EMR Optimisation, CALHN Strategy & Digital, Central Adelaide Local Health Network, SA Health, Adelaide, South Australia Australia; 5 Central Adelaide Local Health Network, SA Health, Adelaide, South Australia Australia; 6 Future Health Systems, College of Health, Adelaide University, Adelaide, South Australia Australia; 7 Department of Neurology, Lyell McEwin Hospital, Northern Adelaide Local Health Network, SA Health, Adelaide, SA, South Australia Australia; 8 Department of Intensive Care, Central Adelaide Local Health Network, SA Health, Adelaide, South Australia Australia; 9 School of Public Health, Adelaide University, Adelaide, South Australia Australia

**Keywords:** Electronic medical records, Hospital resource management, Health information systems, Data quality, Patient flow

## Abstract

**Background:**

Hospitals face increasing strain from rising clinical complexity and demand. Traditional resource allocation approaches often lack the granularity and timeliness needed for responsive planning. This study evaluates whether routinely collected electronic medical record data can be used to classify hospital inpatients into resource-based groups to support real-time planning and hospital-wide operational management.

**Methods:**

A retrospective analysis was conducted on 332,711 inpatient admissions to a quaternary public hospital in South Australia between January 2020 and January 2025. Patients were classified into one of four flow streams within 72 h of admission using a resource-based classification framework developed through a modified Delphi process and validated by clinical review. Summary statistics were used to assess differences in resource use across streams and to evaluate classification stability. Data quality limitations and documentation variability were also assessed.

**Results:**

Flow streams demonstrated distinct differences in length of stay, diagnostic testing, consultations, and allied health input. The model showed strong initial stability, with fewer than 5% of patients changing streams during admission. Key data quality issues included inconsistent consultation documentation, underuse of structured fields, and retrospective overwriting of demographic information, affecting visibility of resource use. Despite these limitations, flow stream classification effectively differentiated patients by resource intensity and care complexity, offering a practical framework to support real-time hospital operations, complementing diagnosis-based groupings.

**Conclusion:**

A structured classification model using routinely collected electronic medical record data can differentiate inpatient resource needs. Flow stream stratification offers a complementary approach to traditional coding-based systems and may help identify operational bottlenecks. With improved documentation and system integration, this approach could enhance hospital responsiveness, resource planning, and overall system performance.

**Supplementary Information:**

The online version contains supplementary material available at 10.1186/s12913-026-14448-8.

## Background

The performance of Australian public hospitals is increasingly challenged by finite funding, rising patient demand, and growing clinical complexity. These pressures contribute to persistent underperformance and capacity constraints across the hospital system, leading to delays in access to care and reduced operational efficiency [1, 2]. 

While electronic medical record (EMR) implementation varies across Australian health services, in hospitals where EMRs are in use, admitted patients are typically assigned to general or subspecialty teams, with most aspects of care documented in the system. In Australia, Diagnosis-Related Groups (DRGs) are retrospectively assigned post-discharge to support hospital reimbursement as part of the national activity-based funding system. While DRGs and similar casemix classification models are well established globally for funding purposes, they offer limited operational utility in real-time and lack prospective insight into resource consumption or patient complexity during admission [3]. EMRs in their current form are largely optimised for documentation, billing reimbursement and regulatory reporting, rather than as dynamic tools designed to support real-time operational decision making [4]. This retrospective focus limits the ability of health services to make timely planning and resource allocation decisions, as these are often informed by lagged data rather than current system demand or patient complexity. Without active visibility of resource consumption and patient needs during admission, healthcare providers face significant challenges in delivering timely, efficient and resource-appropriate care.

The Theory of Constraints (TOC) provides a structured framework for identifying and managing bottlenecks within complex adaptive systems, such as healthcare [5]. By focusing on performance limiting constraints, TOC enables targeted interventions to improve throughput and efficiency contributing to workflow redesign by redirecting patient pathways away from identified bottlenecks resulting from workforce shortages, increased demand for specific services or rigid legacy rule-based models of care. Applied to hospital operations, TOC can facilitate real-time decision-making and resource optimisation, addressing the limitations posed by retrospective data systems like DRGs.

Despite claims that EMR systems can enhance clinical care and support proactive resource planning [6], these benefits have yet to be fully realised in practice [7, 8]. Outcomes remain highly dependent on the operational characteristics and workflow alignment within each healthcare organisation. A disconnect remains between EMR system design and clinical workflows, resulting in limited visibility of system-wide operations and actual resource use [9, 10]. 

We hypothesise that stratifying patients into operationally defined ‘flow streams’ - cohorts grouped by care demands early in their admission- using routinely collected EMR data, could unlock potential to understand service demand in real-time and provide actionable intelligence to guide hospital-wide resource management and improve system efficiency.

The aim of this study was to evaluate whether routinely collected data in the EMR could feasibly be used to classify admitted patients into cohorts based on resource use patterns during early admission.

## Methodology

The study is a retrospective analysis of EMR data of all patients admitted to a quaternary hospital in Adelaide, South Australia (SA) between January 2020 and January 2025. The study consists of three key components: data extraction, creation of new patient flow streams, and descriptive analysis. This study is reported in accordance with the RECORD (Reporting of studies Conducted using Observational Routinely-collected health Data) guidelines [11]. 

### Data

#### Ethics

Approval was granted by the Central Adelaide Local Health Network (CALHN) Human Research Ethics Review Committee (2024/HRE00225), with a waiver of individual consent due to the retrospective nature and scale of the cohort. Access to EMR data was authorised by the data custodian, and data handling complied with relevant governance and privacy legislation.

#### Data source, extraction and internal linkage to capture encounters

Data were extracted from the hospital’s fully integrated Altera Digital Health Sunrise EMR system, using the SA Health Data Analytics Platform, an integrated data infrastructure capable of large-scale interrogation and extraction from the EMR. To accurately represent complete admission episodes, internal deterministic linkage was performed using medical record numbers (MRNs) and time-stamped metadata to consolidate care events (e.g., emergency department visits and inpatient stays) into a unified patient admission journey for each presentation.

A time-based threshold associated related care events. MRNs were subsequently mapped to a study-specific unique patient identifier in a separate, secure mapping file maintained outside the analytical dataset. This approach enabled the dataset to remain fully de-identified during analysis, while preserving the capacity for controlled re-identification. Manual review was conducted for a small number of cases with documentation inconsistencies. Linkage accuracy was verified through sample checks and cross-validation with official admission counts from the health network’s Business Intelligence Unit.

Data extraction and transformation were performed within the SA Health enterprise analytics environment on the Snowflake platform, where extraction logic and analytical code were developed and stored. Extracted datasets were made available for analysis through export to structured spreadsheet files. These files were subsequently loaded into statistical software packages for analysis and assessment. Spreadsheet files were used solely as an intermediary data transfer format and were not used for primary analysis.

#### Data variables

No sampling or exclusion criteria were applied to the admission cohort in this study, to capture the complex operational reality of diverse, dynamic, and evolving nature of hospital resource demands. This comprehensive approach ensures that infrequent but high-impact events, such as seasonal demand surges, unplanned service disruptions, or escalation responses are represented in the dataset. Such inclusivity enables a more accurate understanding of system pressures across routine and exceptional conditions. For example, during periods of severe and sustained access block, classified under the CALHN Demand Escalation Framework (unpublished internal document, Central Adelaide Local Health Network, 2025), real-time admission volumes, resource consumption patterns, and flow stream distribution can now be examined in relation to system strain.

A comprehensive range of variables were extracted from the EMR including demographic details, clinical information such as comorbidities, diagnostics (pathology and imaging) and treatments (medications, procedures, operations, and therapies). Risk assessment data were captured using validated tools for skin integrity [12], cognition [13], malnutrition [14], and mobility [15]. These variables are summarised in Table 1 and detailed further in Appendix 2. Variables were derived from a combination of structured EMR fields and targeted extraction from semi-structured free-text fields. Where possible, measures were obtained from structured or time-stamped data elements, with diagnostic and consult activity quantified using order counts. Targeted free-text extraction was restricted to standardised past medical history (PMHx) fields to support comorbidity identification, while other free-text clinical notes were not systematically extracted due to variability in documentation practices. Full variable definitions and data sources are summarised in Table 1.

In this study resource utilisation was defined as measurable indicators of diagnostic intensity, workforce input, care transitions and bed occupancy that reflect operational demand on hospital services during an admission. The specific resource utilisation variables used are listed in Table 1. These variables were selected based on their routine availability within the EMR, relevance to operational workload and capacity pressure, and their ability to capture variation in care intensity across admissions. To explore the relationship between evolving care needs and classification stability, a convenience sample of cases with clearly observed changes in flow stream allocation were reviewed to assess corresponding shifts in patients care requirements and resource utilisation.

**Table 1:** Summary of Variables Extracted from the Electronic Medical Record (EMR)

Variable	Description	Data Type
Demographic Details	Age	Derived from date of birth (structured field)
	Gender	Structured categorical (EMR field)
	Postcode	Structured numerical (EMR field)
Clinical Information	Comorbidities	Composite: problem list, ICD-10 codes, and targeted PMHx free-text extraction
	Treatment received	Structured categorical (orders/procedures)
	Clinical course	Administrative events (timestamps, orders)
	Discharge disposition	Structured categorical (administrative field)
Risk Assessments	Validated tools for skin integrity (Braden Score [12]), cognition (4AT [13]), malnutrition (MUST [14]), and mobility [15]).	Structured numeric (nursing risk screening)
Diagnostic Information	Blood Tests	Derived count from pathology orders
	CT and MR orders	Orders placed for procedures and derived order counts
	Interventional Radiology orders	Orders placed for procedures and derived order counts
Resource Utilisation Metrics	Allied health* and pharmacy consultation	Structured consult orders
	Sub-specialty consultations	Structured consult orders
	Hospital length of stay	Derived from admission and discharge timestamps
	One to one nursing care	Structured order
	Intra-hospital transfers	Derived count from location timestamps
	Ward type (home vs. outlier)	Structured categorical
	ICU admission	Timestamped ICU entry and exit events
Coding Data	International Classification of Diseases (ICD)-10 codes**	Structured coded field
	DRG Procedure codes [16]	Structured coded field
	DRG Severity codes [16]	Structured coded field

*Allied Health Professional (AHP) considered as Physiotherapy, Occupational Therapy, Speech Pathology, Dietetics and Social Work

** ICD-10 is the 10th revision of the International Classification of Diseases, a medical classification list by the World Health Organization [17].

PMHx: Past medical history; DRG: Diagnostic related group; CT and MR: CT and MRI imaging

#### Data de-identification and cleaning

All patient data were de-identified using a unique study identifier. The dataset was cleaned to ensure consistency, including removal of duplicate entries, standardising formats (e.g., date/time fields), correction of errors, and exclusion of clinically implausible outliers (e.g., negative timestamped data). Missing data were addressed pragmatically: if a variable was not recorded in structured EMR fields, it was assumed not collected. This assumption reflects routine clinical documentation practices and the study’s focus on operational visibility from real-time data. However, this may underestimate some clinical characteristics or care events captured only in free text or omitted due to documentation variability.

#### Data quality assessment

Data quality was assessed to evaluate the completeness, consistency, and operational reliability of key variables prior to analysis. This included verifying demographic information, inconsistencies in service use documentation, and limitations in patient journey mapping across care transitions. Particular attention was given to data elements central to modelling patient flow and resource utilisation such as time-stamped transitions of care, moves to critical service delivery areas for urgent interventions and diagnostics. Front-end clinical documentation was compared with analytics extracts to identify discrepancies and assess the extent to which informal workflows (often recorded in unstructured free-text fields) were missing from the structured EMR data that is used for analysis and planning.

To support this assessment, a stratified sample of 50 patient records per flow stream were manually reviewed by clinicians using full EMR records via the clinical interface (see Appendix 1). These insights informed downstream modelling decisions and guided interpretation of care delivery patterns.

### Creation of new patient flow streams

A novel classification framework was developed to stratify patients into four distinct flow streams based on resource use. The initial framework was developed internally prior to this study using a modified Delphi Technique involving structured consensus from a multidisciplinary panel of expert clinicians with operational and frontline experience. This expert-informed approach ensured that the resulting flow streams were clinically meaningful and grounded in real-word care delivery. During early analysis, the framework was further refined to better reflect observed variation in patient profiles, resulting in four discrete utilisation categories. Table 2 presents the finalised rule set applied to the dataset.

**Table 2:** Flow stream definitions and classification criteria

Flow Stream	Definition	Criteria	Operational Considerations	Opportunities for Future Improvement
1	*Short-stay or ambulatory care patients*	• Day Procedure or Treatment • LOS < 24 h	Operational interest when expected discharge is delayed or procedural outcomes are not achieved.	Refine patient selection protocols to maximise efficiency and minimise avoidable overnight stays.
2	*Admissions for a primary condition under a single clinical specialty*	• ≤ 1 medical consult^1^ • Length of stay < 5 days	Well-suited to care standardisation through clinical pathways.	Optimise clinical workflow and care timeliness through pathway adherence and streamlined delivery.
3	*Patients of intermediate complexity requiring multidisciplinary input*	• Do not meet criteria for FS1, FS2, or FS4	Benefit from early senior clinical decision-making to determine care pathways.	Implement targeted risk stratification and escalation triggers to reduce care delays and prevent deterioration.
4	*Hypercomplex*,* high-acuity admissions requiring intensive resource mobilisation*	• ICU admission ≥ 24 h **OR** • Critical Location – ED Resus, Theatre, ICU, Spinal • Emergency surgery or theatre listed • Treatment Intensity (CT or MR) and ≥ 5 bloods • Group + Hold ordered • Within 24 h: • ≥ 3 medical consults^1^ • ≥ 4 intra-hospital transfers	Benefit from proactive care coordination to manage complexity and mitigate system strain.	Design robust models of care with embedded coordination, workforce agility, and real-time monitoring to manage surge and mitigate systemic disruption.

^1^ Medical consult refers to a documented consultation request to a speciality medical team outside the admitting unit, as recorded in the EMR consult order field

Note: Some criteria (e.g. length of stay < 5 days are assessed dynamically during admission. Patients may be reclassified as their care evolves. For instance, a patient initially meeting Stream 2 criteria may transition to Stream 3 or 4, if their admission exceeds five days or requires additional resources. This dynamic assignment supports real-time planning and operational responsiveness

To minimise misclassification and reduce potential confounding, a structured hierarchical allocation was used to classify patients into flow streams based on observed care delivery patterns. Each admission was retrospectively assessed for eligibility beginning with Flow Stream 1 (FS1), which reflects short-stay, low-resource admissions. If criteria were not met, patients were sequentially evaluated for assignment to Flow Stream 4 (FS4), 2 (FS2), and finally 3 (FS3), based on observed resource intensity and care characteristics. This approach ensured assignment to the highest appropriate resource-intensity stream and prioritised early identification of high-demand cases. While this study focused on retrospective classification using complete admission data, the same logic may be applied at key early points in the patient journey (e.g. 24-72 h) to predict likely stream assignment based on partial data. Patients who did not meet defined criteria for Streams 1, 2, or 4 were allocated to FS3, a heterogeneous group retained for future refinement through machine learning-based classification models.

#### Clinical validation of flow stream allocation

Clinical validation was undertaken by clinicians with relevant medical, nursing and allied health backgrounds on a stratified sample across all flow streams, using full EMR records accessed through the clinical interface. Classification accuracy was assessed by comparing assigned stream categories against actual care pathways and resource use. Discrepant cases were reviewed in multidisciplinary consensus discussions, and findings were used to refine stream definitions and allocation logic. Full methodology and examples are provided in Appendix 1.

### Statistical analysis

Descriptive statistics examined hospital resource utilisation trends at three time points after admission: 24 h, 72 h, and at discharge. This enabled assessment of transitions between flow streams or shifts in resource-use profiles over the course of an admission.

Descriptive characteristics, including age, gender, medication burden, and list of investigations, were analysed and stratified across flow streams to identify differences in patient complexity and care patterns.

Summary measures included medians and interquartile ranges of key resource variables, as these are more appropriate than means for data with skewed distributions and operational outliers, such as length of stay and consult counts. Comparative analysis across flow streams was undertaken to evaluate the alignment between anticipated and actual resource intensity, informing iterative refinement of stream definitions and identification of potentially misclassified or transitional cases. For example, the original FS2 criteria limited patients to a single sub-specialty consult; however, analysis identified a subset of short-stay patients who received a second consult from their usual treating team to support continuity of care. In response, the classification rule was modified to allow up to two consults in FS2.

Statistical analyses were performed using R, version 4.4.3 and STATA, version 18.5. The summary statistics in Table 3 were generated with the R package, gtsummary [18]. 

## Results

**Fig. 1:**
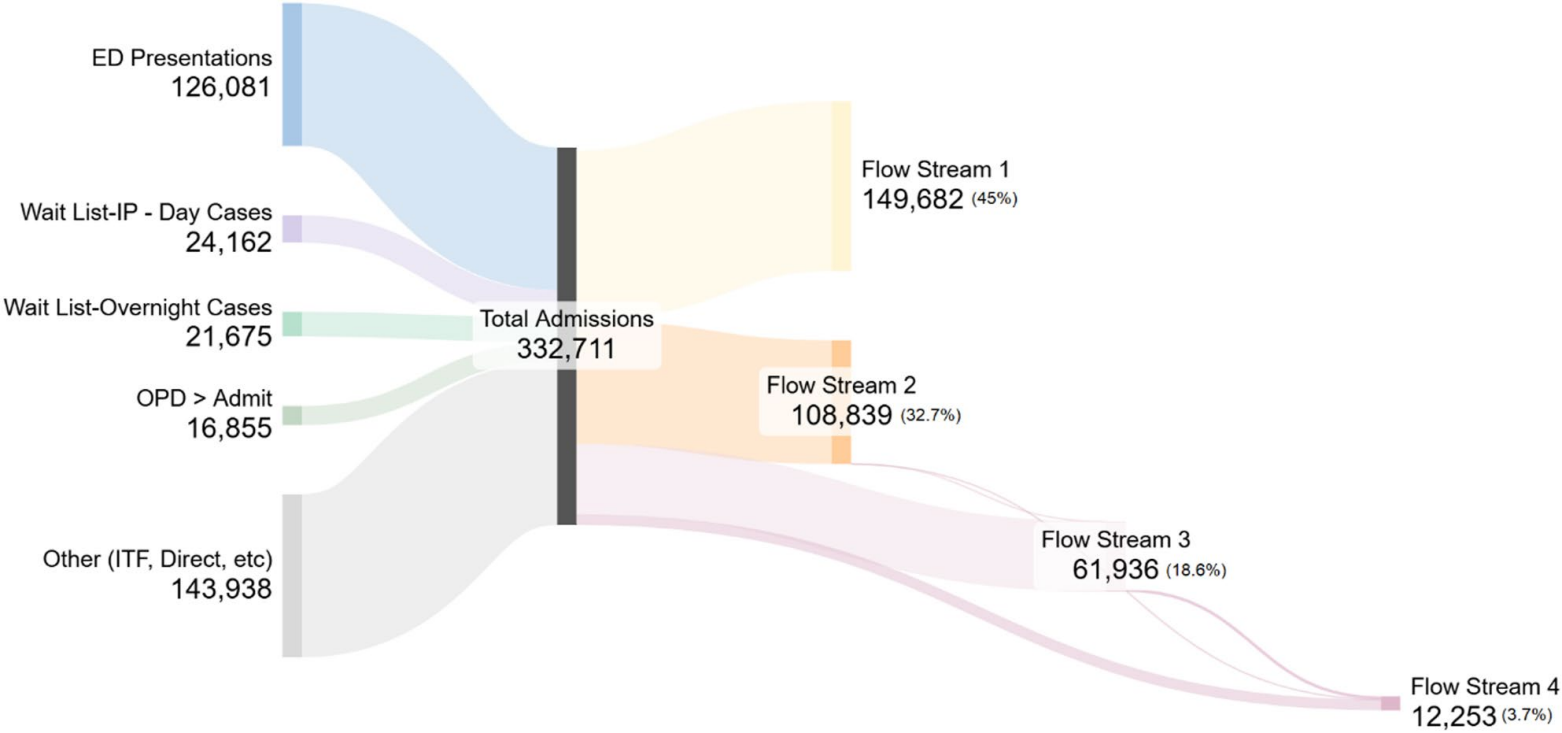
Distribution of inpatient admissions by source and final Flow Stream classification. ED: Emergency Department; OPD: Outpatient Department; IP: Inpatient, ITF: Inter-hospital Transfer

**Fig. 2:**
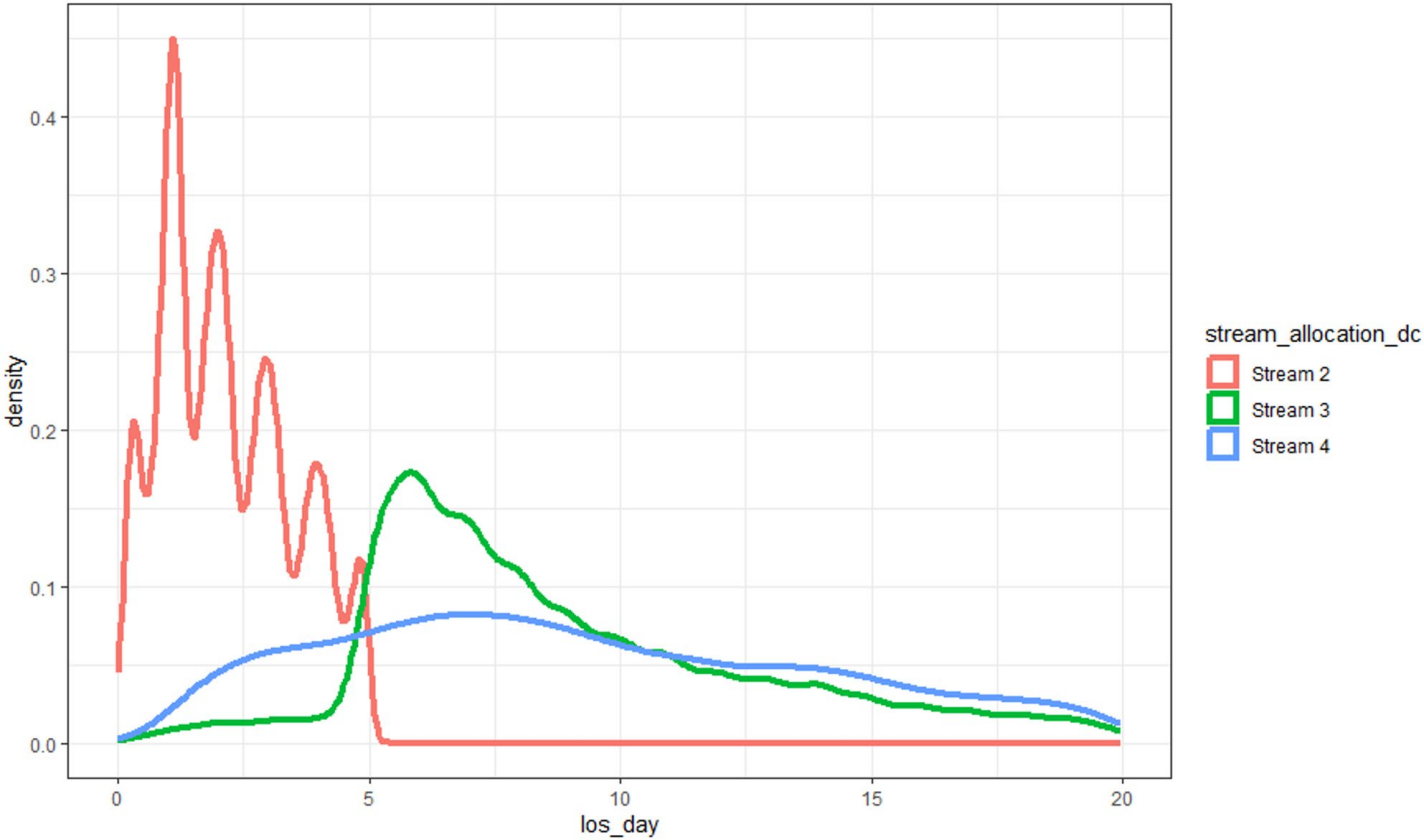
Length of stay distribution stratified by flow stream* *All FS1 LOS = 1 day or less

Between January 2020 and January 2025, a total of 332,711 inpatient admissions were recorded. The distribution of these admissions by source and final flow stream classification is shown in Fig. 1. A high-level summary is presented in Table 3, with full descriptive statistics provided in Appendix 2. Patterns of hospital length of stay by flow stream, also shown in Table 3, are visualised in Fig. 2 to highlight distributional differences across Streams 2-4. Stream 1 is excluded due to its consistently short and tightly clustered length of stay (median 0.2 days), which presents as a near zero flat line and limits interpretability. These variables informed flow stream classification and supported early assessment of hospital resource distribution.

All findings presented in this section are descriptive and unadjusted, consistent with the exploratory objectives of this study’s initial phase. No statistical modelling or hypothesis testing was performed, as the focus was on classification accuracy and resource profiling.

**Table 3:** Summary characteristics of the total study population, stratified by flow stream

Variable	Overall *N* = 332,711	Stream 1 *N* = 149,682	Stream 2 *N* = 108,840	Stream 3 *N* = 61,936	Stream 4 *N* = 12,253
**Age**,** years**^**1**^	63 (48, 75)	61 (48, 72)	62 (44, 77)	70 (56, 81)	62 (49, 73)
**Female**,** n (%)**	139,733 (42.0)	59,799 (40.0)	47,750 (44.0)	27,548 (44.0)	4,636 (38.0)
**Length of Stay**,** days**^1^	0.9 (0.2, 4.0)	0.2 (0.2, 0.3)	2.0 (1.1, 3.1)	8.4 (6.2, 13.3)	11.2 (6.4, 20.3)
**Admission Type**^**2**^, **n (%)**					
Medical	204,286 (61.0)	108,437 (72.4)	53,338 (49.0)	37,144 (60.0)	5,367 (43.8)
Surgical	113,982 (34.0)	28,027 (18.7)	54,471 (50.0)	24,607 (39.7)	6,877 (56.2)
Other^3^	14,443 (4.3)	13,218 (8.8)	1,031 (0.9)	185 (0.3)	9 (< 0.1)

^1^ Median (interquartile range)

^2^Admission Type – Mental Health admissions were excluded from this analysis

^3^Other: Emergency, Hyperbaric, Gynaecology

### Data quality assessment

Evaluation of EMR data quality revealed substantial limitations in the completeness and standardisation of critical data fields. While admission note completion rates were high within 72 h, structured fields were often bypassed in favour of free-text documentation, limiting downstream extractability. High variability in documentation practises was noted across clinical teams and disciplines, contributing to inconsistent data capture and reducing the reliability of certain structured fields for operational analysis. Mandatory fields were frequently populated with inaccurate or generic responses, for example, patients admitted from residential aged care were commonly recorded as arriving from private residences.

Structured problem lists, intended to support longitudinal care continuity, were populated in less than 75% of cases. High-acuity events such as the massive transfusion pack (MTP) activation were significantly underreported in the EMR compared to external validation sources (e.g., 144 EMR entries in total cohort versus 355 blood bank activations in a single year). Consult orders were inconsistently used across clinical teams, particularly in urgent settings where informal referrals and verbal communication were preferred over electronic pathways. In some cases, consults were entered by the admitting team for their own service as part of the admission process, which distorted flow stream allocation. Due to inconsistencies in naming conventions between consult orders and admitting team identifiers, manual coding was necessary to accurately match and exclude these entries for valid classification (see Appendix 2 for variable-level missing data).

No linkage failures were identified in the final dataset. Manual validation of a subset of patient records confirmed concordance between administrative admission identifiers and consolidated care episodes, supporting the integrity of the linkage process.

### Flow stream assignment

**Fig. 3:**
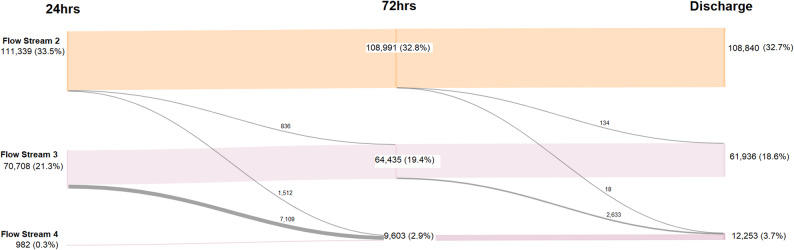
Flow stream transitions at 24 h, 72 h, and discharge. Note: Node labels show number and percentage of total admissions (*N* = 332,711). Numbers adjacent to transition paths indicate the number of patients reclassified between flow streams. Flow Stream 1 is not shown; transitions involving FS1 are described in the accompanying text

All eligible admissions were classified into one of four resource-based flow streams using the allocation framework. The results are summarised in Table 4, comparing the initial projected distribution of patients across streams with actual classifications at 24 h, 72 h and discharge, allowing assessment of classification stability and operational alignment with early admission profiles. As the model reflects resource utilisation, patient allocation may shift during admission in response to changes in care intensity or clinical deterioration, a characteristic designed to support real-time operational insight, as visualised in Fig. 3.

**Table 4:** Flow stream classification of admissions at 24 h, 72 h, and discharge (*N* = 332,711)

Flow Stream	24 h, *n* (%)	72 h, *n* (%)	Discharge, *n* (%)
FS1	149,682 (45.0)	149,682 (45.0)	149,682 (45.0)
FS2	111,339 (33.5)	108,991 (32.8)	108,840 (32.7)
FS3	70,708 (21.3)	64,435 (19.4)	61,936 (18.6)
FS4	982 (0.3)	9,603 (2.9)	12,253 (3.7)

Note: Percentages are based on total admissions (*N* = 332,711) at each time point. Values are rounded to one decimal place for clarity

Fewer than 5% of patients changed flow stream assignment during admission. These changes predominantly involved transitions from FS3 to FS4 as clinical complexity emerged, and from FS2 to FS4 in cases requiring escalation to higher levels of care. This pattern is evident in the transition flow visualised in Fig. 3, where movement from FS2 and FS3 to FS4 accounts for most reclassifications. For example, a patient initially classified as FS2 due to a stable embolic stroke presentation was later reclassified as FS4 following rapid clinical deterioration, requiring ICU admission and neurosurgical intervention.

Transitions out of FS1 are not visualised in the same way due to the nature of this stream. FS1 is designed for patients admitted for low-complexity, predictable, scheduled care. When these patients experience unplanned escalation or clinical deterioration, they are reclassified directly into FS2, FS3 and FS4 based on resource intensity and complexity, rather than transitioning within FS1. As such, FS1 serves a distinct entry stream rather than a trajectory within the flow stream model.

This low reallocation rate suggests strong initial model stability and highlights stream transitions are not frequent, instead representing meaningful changes in patient condition or care requirements. Establishing baseline ratios of the flow stream is critical to evaluating responses to external and internal system characteristics over time, to allow changes to resource reallocation or other strategic interventions.

### Operational utility of flow stream classification

Initial application of the classification model revealed clear differentiation in resource-use profiles across streams. FS1 patients followed short-stay, low-resource pathways, with minimal investigations or consults, a median LOS of 0.2 days, rare ICU admission (3.1%) and polypharmacy in 0.6% of cases. FS2 admissions remained within specialty teams with relatively short lengths of stay (median 2.0 days) and focused care delivery, with low allied health input (median 0 (0,1)). FS3 captured patients of intermediate complexity shown by median LOS of 8.4 days, 21% requiring multidisciplinary input from medical and allied health. Polypharmacy was high (93%) and ICU admission was greater than FS1 and FS2. FS4 admissions represented high acuity care: 96% were admitted to ICU, 36% had multiple specialist consultations and the median length of stay was 11.2 days.

## Discussion

This retrospective observational study evaluated the operational utility of a novel, resource-based patient classification model using routinely collected EMR data. The study aimed to determine whether structured EMR variables could reliably stratify patients into flow streams reflective of care complexity and resource use to inform hospital-wide resource allocation. The analysis included inpatient admissions (*n* = 332,711) to a quaternary hospital in South Australia between January 2020 and January 2025. While the model was developed in an Australian hospital, the operational challenges addressed, such as managing resource-intensive cohorts, identifying system bottlenecks, and supporting real-time planning, are shared across many hospital systems globally. The framework is designed to be adaptable to local infrastructure, EMR capabilities, and policy environments.

A comprehensive dataset was constructed through deterministic linkage and rigorous data cleaning, incorporating demographic, clinical, diagnostic, and operational variables. Patients were stratified into one of four flow streams based on resource utilisation rather than diagnostic grouping. Despite limitations in structured EMR documentation, particularly variability in consult orders and data entry, the model demonstrated early utility. Flow streams captured distinct care complexity and service utilisation patterns, which remained stable over the inpatient stay [8]. Fewer than 5% of patients changed stream assignment during admission, suggesting allocation stability and that transitions likely reflect meaningful clinical changes.

By grounding classification in observed resource consumption, this model provides a structured framework that could support dynamic, real-time operational planning if implemented prospectively. In line with Theory of Constraints (TOC) principles, the framework demonstrates the capacity to identify resource-intensive cohorts and potential operational bottlenecks early in an admission, informing system responsiveness for staffing, bed management, and escalation planning, before broader strain emerges. However, because flow stream assignment in this study was derived retrospectively using complete admission data, the direct operational benefits described here are not yet realised in practice and would only be fully achieved when stream allocation can be predicted or determined early in the admission, ideally at or shortly after presentation. While this evaluation is retrospective, the stability and differentiation observed between flow streams suggest the approach is suitable for future real-time application. The broader value of the model lies in its potential to support continuous operational insight and pre-emptive decision making, while avoiding the unintended consequence of directing additional workload toward already strained services.

Many hospital performance constraints are internally generated, arising from how care delivery is structured, prioritised, and resourced. High resource patients in FS4, for instance, exert disproportionate demand on critical care infrastructure, which can delay care delivery and limit access for lower-acuity patients [19] in FS1, FS2 and FS3. This cascading effect highlights the need to monitor high-acuity cohorts to mitigate system-wide impacts. TOC asserts that inefficiencies stem from unidentified or unmanaged constraints, which lead to cascading delays, access blocks, and disproportionate demands on high-acuity services. These constraints are not static and can be mitigated through targeted redesign and strategic resource realignment. The flow stream model offers a mechanism to identify operational pressure points by stratifying patients according to resource demand, supporting both direct benefits, such as early identification of capacity limits, and indirect benefits, including improved patient flow for other cohorts. These dynamics are especially relevant during periods of high occupancy, where marginal increases in volume can lead to disproportionate delays, reduced flexibility and compromised overall system efficiency. By anticipating pressure points and supporting proactive resource alignment, the model may help sustain performance under demand stress. Without such visibility, health services risk reinforcing inefficiencies by directing demand to constrained areas, inadvertently exacerbating stress where there is least capacity. Embedding this model into real-time operational planning may improve system resilience and performance at scale.

A critical next step is evaluating how the distributions shift over time in response to internal or external pressures. For example, a rise in FS4 admissions may signal emerging strain. These patients often bypass routine scheduling, displacing lower-acuity patients and contributing to delays, blocked beds, and inefficiencies across the hospital. Monitoring these shifts in near real-time could support decision-makers in enacting timely interventions, reallocating resources, or adjusting discharge pathways before system-wide dysfunction occurs.

Although EMRs are structured to standardise data capture and support visibility, the reality of frontline practice often diverges. Critical workflows remain undocumented due to time pressures, perceived irrelevance of fields, or the convenience of informal workarounds. Clinicians bypass digital systems in favour of direct communication (e.g., phone calls) or offline tools (e.g., spreadsheets), which, while efficient in context, obscure visibility at the organisational level. This gap between system design and care delivery impairs the ability to learn from frontline adaptations [20], which carries significant operational risks. Planning, funding, and workforce forecasting frameworks increasingly rely on EMR-derived data, yet these decisions are compromised by inconsistencies in data entry, underutilisation of structured fields, and underreporting of resource-intensive activity [21]. While technically available, structured fields are often bypassed due to poor integration with clinical workflows and a lack of demonstrated value for accurate documentation, to provide system-level insight and responsiveness [21]. The absence of reliable visibility at scale impairs the organisation’s ability to anticipate demand, respond to emergent complexity, measure care delivery and allocate resources intelligently. To support adoption, decision tools must be embedded into clinicians’ natural workflow rather than additional external overlays. Operationally relevant nudges, and prioritisation prompts, could be integrated directly into the EMR to drive meaningful use [22]. 

These challenges also intersect with important policy and ethical considerations. As the study progresses toward predictive analytics and machine learning applications, the ethical integration of such models into clinical and operational practice must be prioritised. Attention to bias mitigation, model transparency [23] and stakeholder engagement (including clinicians, executives and consumers), will be essential in guiding methodological refinement and governance.

This framework offers a structured approach to developing real-time operational stratification using routinely collected EMR data. By monitoring complexity and care intensity over time, the model demonstrates how EMR-derived information can be organised to support a more adaptive, data-driven approach to hospital-wide resource management. Much of the existing literature on EMR data use emphasises its secondary application in retrospective research, population health, and analytics for outcomes or epidemiologic studies rather than real-time operational classification or resource planning [24]. The flow stream model developed will serve as the foundation for machine learning-based predictive analytics in the next phase, aimed at near real-time decision making and surge response. Future phases could identify delay points, such as prolonged ED stays or diagnostic bottlenecks, and support earlier escalation, targeted interventions, and more responsive resource alignment. This transition from retrospective classification to early prediction is essential for translating the model’s analytical insights into tangible operational impact.

## Limitations

This study has several limitations. First, it is based on retrospective EMR data, which is inherently subject to constraints in completeness, accuracy, and consistency of historical documentation. Where structured data were missing, it was assumed that the information had not been recorded during the admission. No imputation was used to preserve record integrity and reflect real-world availability. While aligned with operational practice, this may have underestimated the frequency of some resource utilisation events, particularly those captured only in free-text notes or omitted entirely due to documentation variability. These limitations are well-recognised in real-world data, where incomplete and heterogeneous data collection can limit interpretability, to reliably analyse, contextualise and apply to operational decision making [25]. 

Second, the analysis was conducted at a single quaternary hospital and may not be generalisable to settings with different infrastructure, case mix, staffing or EMR capability. Third, some components of care remain inconsistently documented in structured EMR fields, particularly in high-acuity or multidisciplinary environments. Verbal referrals, Medical Emergency Team (MET) calls, and non-standardised documentation of specialist consult input were frequently observed in unstructured notes, leading to an underestimation of resource use and complexity.

Additionally, a structural limitation of the EMR system relates to demographic fields that are stored at the client level rather than at the individual admission level. Variables such as usual accommodation and postcode are retrospectively overwritten across prior admissions if updated during a later encounter. For example, if a patient was admitted from a private residence early in the study period but subsequently moved to a residential aged care facility, all historical admissions may be updated to reflect the latter. While this has limited impact on real-time model development, it introduces a source of temporal inaccuracy for retrospective review and flow stream assignment. In the absence of audit trail access for these fields, this limitation reflects a broader challenge in the underlying EMR data architecture.

Finally, this study presents descriptive findings only. While the model demonstrates early operational value, it does not yet incorporate real-time analytics or predictive capabilities. These will be developed and evaluated in subsequent phases of the study.

## Conclusion

This study introduces a novel, resource-based flow stream framework to better classify patients by resource use rather than diagnosis. The model has operational relevance for hospital planning and performance monitoring but is constrained by EMR documentation gaps that limit visibility of care delivery. System inefficiencies are compounded by informal workflows and inconsistent structured data use, creating hidden constraints. From a TOC perspective, these bottlenecks restrict system throughput and remain unaddressed without real-time data insight.

Future work must focus on strengthening real-time analytics, embedding decision support into EMR workflows, and developing predictive tools grounded in clinical and operational practice. This study forms the foundation for a multi-year evaluation of EMR adaptability, with the next phase focused on near real-time identification of bottlenecks and AI-driven forecasting to support smarter resource allocation. Aligning data with action is essential to building a learning health system that enables agile, proactive hospital operations.

## Supplementary Information

Below is the link to the electronic supplementary material.

Supplementary Material 1

Supplementary Material 2

## Data Availability

The datasets generated and/or analysed during the current study are not publicly available due to institutional and legal data access restrictions. De-identified data may be made available upon reasonable request and subject to appropriate ethics and governance approvals from SA Health and the Central Adelaide Local Health Network.

## Abbreviations

4AT: 4 ‘A’s Test (Delirium Screening Tool)
AHP: Allied Health Professional
AI: Artificial Intelligence
CALHN: Central Adelaide Local Health Network
CT: Computed Tomography
DRG: Diagnosis-Related Group
ED: Emergency Department
EMR: Electronic Medical Record
FS: Flow Stream
HREC: Human Research Ethics Committee
ICD-10: International Classification of Diseases, 10th Revision
ICU: Intensive Care Unit
LOS: Length of Stay
MDC: Major Diagnostic Category
MET: Medical Emergency Team
MR: Magnetic Resonance
MRN: Medical Record Number
MTP: Massive Transfusion Pack
MUST: Malnutrition Universal Screening Tool
PET: Positron Emission Tomography
PMHx: Past Medical History
RAH: Royal Adelaide Hospital
RECORD: REporting of studies Conducted using Observational Routinely collected Data
SA: South Australia
TOC: Theory of Constraints

## References

[1] Australian Medical Association. Public hospitals in logjam as funding pressures grow. 2023. https://ama.com.au (accessed 19 Aug 2025).

[2] Duckett S, Breadon P. Controlling costs and improving care: reforming the hospital funding system. Grattan Institute; 2014.

[3] Jackson T, Michel JL, Roberts RF, et al. A classification of hospital admissions. BMC Health Serv Res. 2014;14:346.25128468

[4] Shen Y, Yu J, Zhou J, Hu G. Twenty-five years of evolution and hurdles in electronic health records and interoperability in medical research: comprehensive review. J Med Internet Res. 2025;27:e59024. 10.2196/59024. 39787599 10.2196/59024 PMC11757985

[5] Almeida MA, Marinho MMO. Theory of constraints in healthcare: a systematic literature review. Int J Qual Reliab Manag. 2022;39(3):716–37.

[6] Yadav S, Kumar R, Tran T, et al. Assessing the predictive and analytics capability of electronic medical records for operational planning. J Healthc Inf Res. 2023;7(2):123–35.

[7] Zozus MN, Stansbury DW, Raskin S, et al. Assessing data quality for healthcare systems. eGEMs. 2019;7(1):20.31106226

[8] Reimer AP, Milinovich A, Madigan EA. Data quality assessment framework to assess electronic medical record data for use in research. Int J Med Inf. 2016;90:40–7. 10.1016/j.ijmedinf.2016.03.006 PMC4845906 27103196

[9] McGinn CA, Gagnon MP, Shaw N, et al. Users’ perspectives of key factors to implementing electronic health records in Canada. JMIR Med Inf. 2011;13(3):e73. 10.1186/1472-6947-12-105 PMC3470948 22967231

[10] Ajami S, Bagheri-Tadi T. Barriers for adopting electronic health records (EHRs) by physicians. Acta Inf Med. 2013;21(2):129–34. 10.5455/aim.2013.21.129-134 PMC3766548 24058254

[11] Benchimol EL, Smeeth L, Guttmann A, et al. The reporting of studies conducted using observational routinely-collected health data (RECORD) statement. PLoS Med. 2015;12:e1001885. 26440803 10.1371/journal.pmed.1001885 PMC4595218

[12] Braden B, Bergstrom N. A conceptual schema for the study of the etiology of pressure sores. Rehabil Nurs. 1987;12(1):8–12. 3643620 10.1002/j.2048-7940.1987.tb00541.x

[13] Bellelli G, Morandi A, Davis DHJ, et al. Validation of the 4AT, a new instrument for rapid delirium screening: a study in 234 hospitalised older people. Age Ageing. 2014;43(4):496–502. 24590568 10.1093/ageing/afu021 PMC4066613

[14] Elia M. The ‘MUST’ report: nutritional screening of adults: a multidisciplinary responsibility. Redditch, UK: BAPEN; 2003.

[15] SA Health. Fall injury and prevention clinical guideline: screening, assessment, care planning and discharge planning. Government South Australia 2018. https://www.sahealth.sa.gov.au/…. (Accessed 19 Aug 2025).

[16] Independent Health and Aged Care Pricing Authority. AR-DRG Version 11.0. https://www.ihacpa.gov.au/resources/ar-drg-version-110 (accessed 27 Aug 2025).

[17] World Health Organization. *International Classification of Diseases, 10th revision (ICD-10)*. https://www.who.int/classifications/icd/en/

[18] Sjoberg D, Whiting K, Curry M, et al. Reproducible summary tables with the gtsummary package. R J. 2021;13:570–80. 10.32614/RJ-2021-053.

[19] Eriksson CO, Stoner RC, Eden KB, Newgard CD, Guise JM. The association between hospital capacity strain and inpatient outcomes in highly developed countries: a systematic review. J Gen Intern Med. 2017;32(6):686–96. 10.1007/s11606-016-3936-3. 27981468 10.1007/s11606-016-3936-3 PMC5442002

[20] Verhagen MJ, de Vos MS, Sujan M, Hamming JF. The problem with making Safety-II work in healthcare. BMJ Qual Saf. 2022;31(5):402–8. 10.1136/bmjqs-2021-014396. 35304422 10.1136/bmjqs-2021-014396

[21] de Groot K, de Bruijne M, Paans W, et al. Effective and feasible interventions to improve structured EHR data quality: a systematic review. Int J Med Inf. 2023;174:105050.

[22] Alexiuk M, Ashcroft R, Pijl-Zieber E, et al. Clinical decision support tools in the electronic medical record. Kidney Int Rep. 2023;8(9):929–38. 38312784 10.1016/j.ekir.2023.10.019 PMC10831391

[23] Sendak MP, D’Arcy J, Kashyap S, et al. Clinical implementation of predictive models embedded within electronic health records. J Am Med Inf Assoc. 2020;27(4):639–46.

[24] Casey JA, Schwartz BS, Stewart WF, Adler NE. Using electronic health records for population health research: a review of methods and applications. Annu Rev Public Health. 2016;37:61–81. 10.1146/annurev-publhealth-032315-021353. 26667605 10.1146/annurev-publhealth-032315-021353 PMC6724703

[25] Weiskopf NG, Weng C. Methods and dimensions of electronic health record data quality assessment: enabling reuse for clinical research. J Am Med Inf Assoc. 2013;20(1):144–51. 10.1136/amiajnl-2011-000681 PMC3555312 22733976

