# Supplementary material for: Can routinely collected electronic medical record (EMR) data support hospital resource allocation? A retrospective analysis of 332,711 presentations to a public quaternary teaching hospital in South Australia (2020–2025)
Source: BMC Health Serv Res. 2026 Mar 27;26:643. doi: 10.1186/s12913-026-14448-8 (PMC13147598; doi:10.1186/s12913-026-14448-8)
Supplement: Supplementary file 1 — Supplementary Material 1 [file 12913_2026_14448_MOESM1_ESM.docx]

**APPENDIX 1. Clinical Validation of Flow Stream Classification**

A clinical validation process was undertaken to assess the accuracy of flow stream assignments against observed patterns of care. A stratified sample of approximately 50 cases per stream was reviewed by a multidisciplinary clinical team, using the full EMR interface to examine documentation, investigations, consults, referrals, and clinical notes.

The validation focused on:

- Concordance between assigned stream and actual resource intensity
- Discrepancies due to missing or inconsistently recorded data
- Adequacy of allocation criteria for capturing patient complexity

Most classifications were found to be appropriate, particularly for Flow Streams 1 and 2, where structured data closely aligned with care delivered. However, discrepancies were more frequent in Flow Stream 4, where:

- Critical events such as Medical Emergency Team (MET) calls, unplanned ICU admissions, and escalation pathways were not captured in structured EMR fields
- Consult orders were inconsistently used, with some entered retrospectively or bypassed entirely
- Allied health involvement and complex discharge planning were often recorded only in free-text progress notes, limiting analytic visibility

One example involved a patient admitted with a stable embolic stroke initially classified as FS2. Following clinical deterioration, ICU transfer, and neurosurgical intervention, the case was reclassified as FS4. This highlighted the importance of capturing emergent complexity during admission.

Insights from the validation process informed targeted revisions to the classification logic, including:

- Clarification of consult thresholds (e.g. inclusion of dual-team review)
- Adjustment of FS4 criteria to account for escalation evidence in unstructured documentation
- Refinement of high-risk diagnostic flags

These changes were applied consistently to similar case types to improve classification accuracy and support broader model generalisability.
