# Supplementary material for: Can routinely collected electronic medical record (EMR) data support hospital resource allocation? A retrospective analysis of 332,711 presentations to a public quaternary teaching hospital in South Australia (2020–2025)
Source: BMC Health Serv Res. 2026 Mar 27;26:643. doi: 10.1186/s12913-026-14448-8 (PMC13147598; doi:10.1186/s12913-026-14448-8)
Supplement: Supplementary file 2 — Supplementary Material 2 [file 12913_2026_14448_MOESM2_ESM.docx]

**APPENDIX 2**

**Table 3. Characteristics of the Study Population, by Flow Stream^1^**

| **Variable** | **N** | **Stream 1**  N = 149,682 | **Stream 2**  N = 108,840 | **Stream 3**  N = 61,936 | **Stream 4**  N = 12,253 |  |
| --- | --- | --- | --- | --- | --- | --- |
| **Age, years^2^** | 332,711 | 61 (48, 72) | 62 (44, 77) | 70 (56, 81) | 62 (49, 73) |  |
| **Female, n (%)** | 332,711 | 59,799 (40) | 47,750 (44) | 27,548 (44) | 4,636 (38) |  |
| **Ethnicity, n (%)** | 332,711 |  |  |  |  |  |
| Aboriginal and/or TSI |  | 11,026 (7.4) | 6,536 (6.0) | 3,465 (5.6) | 1,038 (8.5) |  |
| Not Aboriginal-TSI |  | 133,604 (89) | 98,694 (91) | 56,267 (91) | 10,606 (87) |  |
| Not Stated |  | 5,052 (3.4) | 3,610 (3.3) | 2,204 (3.6) | 609 (5.0) |  |
| **Residence Location, n (%)** | 332,181 |  |  |  |  |  |
| Local |  | 63,497 (42) | 44,160 (41) | 24,976 (40) | 3,652 (30) |  |
| Other Adelaide Metro |  | 52,877 (35) | 30,775 (28) | 17,015 (28) | 3,702 (30) |  |
| Rural SA |  | 30,278 (20) | 29,448 (27) | 17,192 (28) | 4,026 (33) |  |
| Outside SA/Unknown |  | 2,906 (1.9) | 4,217 (3.9) | 2,629 (4.3) | 831 (6.8) |  |
| Missing, n |  | 124 | 240 | 124 | 42 |  |
| **Admission Type^3^, n (%)** | 332,711 |  |  |  |  |  |
| Medical |  | 108,437 (72) | 53,338 (49) | 37,144 (60) | 5,367 (44) |  |
| Surgical |  | 28,027 (19) | 54,471 (50) | 24,607 (40) | 6,877 (56) |  |
| Other^4^ |  | 13,218 (8.8) | 1,031 (0.9) | 185 (0.3) | 9 (<0.1) |  |
| **Length of Stay, days^2^** | 332,707 | 0.2 (0.2, 0.3) | 2.0 (1.1, 3.1) | 8.4  (6.2, 13.3) | 11.2  (6.4, 20.3) |  |
| **Number of Blood Tests^2^** | 332,711 | 0 (0, 0) | 6 (3, 9) | 10 (7, 14) | 14 (11, 19) |  |
| **Home Medication Count^2^** | 110,522 | 10 (7, 14) | 8 (4, 12) | 9 (5, 13) | 6 (3, 10) |  |
| Missing, n |  | 149,117 | 62,400 | 8,937 | 1,735 |  |
| **Polypharmacy^5^, n (%)** | 227,091 | 322 (0.6) | 59,974 (59) | 55,544 (93) | 11,617 (98) |  |
| Missing, n |  | 94,977 | 7,819 | 2,392 | 432 |  |
| **Number of Imaging Requests^2^** | 332,711 | 0 (0, 0) | 0 (0, 1) | 1 (0, 2) | 1 (0, 3) |  |
| **Any Advanced Imaging, n (%)** | 332,711 | 860 (0.6) | 38,282 (35) | 40,739 (66) | 9,043 (74) |  |
| **Any Interventional Radiology, n (%)** | 332,711 | 404 (0.3) | 2,058 (1.9) | 3,724 (6.0) | 1,739 (14) |  |
| **Any ICU Admission, n (%)** | 332,711 | 4,663 (3.1) | 2,041 (1.9) | 3,075 (5.0) | 11,736 (96) |  |
| **≥2 Medical Consultations, n (%)** | 332,711 | 2 (<0.1) | 0 (0) | 13,219 (21) | 4,390 (36) |  |
| **Allied Health Consult Count^2^** | 332,711 | 0 (0, 0) | 0 (0, 1) | 2 (1, 3) | 2 (1, 4) |  |
| **1:1 Nursing Order Placed, n (%)** | 332,711 | 1 (<0.1) | 828 (0.8) | 1,488 (2.4) | 477 (3.9) |  |
| **Last Disposition, n (%)** | 332,711 |  |  |  |  |  |
| Home |  | 144,638 (97) | 88,044 (81) | 33,195 (54) | 5,947 (49) |  |
| Other Hospital or Healthcare Facility |  | 303 (0.2) | 6,476 (6.0) | 16,167 (26) | 3,732 (30) |  |
| Hospital in the Home |  | 708 (0.5) | 2,983 (2.7) | 2,881 (4.7) | 416 (3.4) |  |
| Residential Aged Care (new) |  | 3 (<0.1) | 177 (0.2) | 1,084 (1.8) | 47 (0.4) |  |
| Residential Aged Care (returning) |  | 3,847 (2.6) | 5,644 (5.2) | 3,592 (5.8) | 106 (0.9) |  |
| Administrative Discharge |  | 16 (<0.1) | 830 (0.8) | 1,563 (2.5) | 263 (2.1) |  |
| Died |  | 79 (<0.1) | 2,470 (2.3) | 2,780 (4.5) | 1,473 (12) |  |
| Absconded/Self Discharged |  | 81 (<0.1) | 2,190 (2.0) | 662 (1.1) | 163 (1.3) |  |
| Other |  | 7 (<0.1) | 26 (<0.1) | 12 (<0.1) | 106 (0.9) |  |
| **Intra-hospital Moves^2^** | 332,631 | 0 (0, 1) | 5 (3, 6) | 6 (4, 8) | 7 (5, 10) |  |
| Missing, n |  | 0 | 61 | 19 | 0 |  |
| **MUST Score = 0, n (%)** | 108,276 | 5,088 (87) | 45,942 (79) | 25,343 (67) | 4,353 (62) |  |
| Missing, n |  | 143,859 | 50,996 | 24,294 | 5,286 |  |
| **Highest Non-zero MUST Score^2^** | 27,550 | 1.00 (1, 2) | 2.00 (1, 2) | 2.00 (1, 2) | 2.00 (1, 2) |  |
| Missing, n | |  | 148,947 | 96,938 | 49,637 | 9,639 |
| **Delirium Score = 0, n (%)** | 122,794 | 10,022 (86) | 42,896 (75) | 26,791 (57) | 4,465 (59) |  |
| Missing, n |  | 137,971 | 51,896 | 15,327 | 4,723 |  |
| **Highest Non-zero Delirium Score^2^** | 38,620 | 1.00 (1, 2) | 2.00 (1, 4) | 3.00 (1, 6) | 3.00 (1, 6) |  |
| Missing, n |  | 147,993 | 94,792 | 42,118 | 9,188 |  |
| **Top 5 Major Diagnostic Categories (MDC), n (%)^6^** | 111,527 |  |  |  |  |  |
| Kidney & Urinary Tract |  | 28,887 (54) | 2,451 (7.4) | 1,204 (5.7) | 138 (3.2) |  |
| Circulatory System |  | 3,209 (6.0) | 4,883 (15) | 2,299 (11) | 904 (21) |  |
| Nervous System |  | 2,098 (3.9) | 3,291 (10.0) | 2,768 (13) | 628 (14) |  |
| Musculoskeletal Sys & Conn Tissue |  | 2,095 (3.9) | 3,277 (10.0) | 3,042 (14) | 269 (6.2) |  |
| Respiratory System |  | 245 (0.5) | 3,342 (10) | 2,496 (12) | 447 (10) |  |
| Missing, n |  | 96,500 | 75,906 | 40,888 | 7,890 |  |
| **DRG Severity, n (%)** | 111,527 |  |  |  |  |  |
| A (Major) |  | 2,241 (4.2) | 9,722 (30) | 12,114 (58) | 2,524 (58) |  |
| B (Intermediate) |  | 16,869 (32) | 17,607 (53) | 6,963 (33) | 1,282 (29) |  |
| C (Minor) |  | 6,472 (12) | 4,364 (13) | 1,741 (8.3) | 453 (10) |  |
| D (Minor) |  | 0 (0) | 134 (0.4) | 2 (<0.1) | 4 (<0.1) |  |
| Z (Unclassified) |  | 27,600 (52) | 1,107 (3.4) | 228 (1.1) | 100 (2.3) |  |
| Missing, n |  | 96,500 | 75,906 | 40,888 | 7,890 |  |
| ^¹^ Not all variables were available for the full study cohort. Ns are shown per row.  ^2^Median (Interquartile range)  ^3^Admission Type – Mental Health admissions were excluded from this analysis.  ^4^Other: Emergency, Hyperbaric, Gynaecology  ^5^Polypharmacy defined as ≥8 home medications  ^6^Top 5 MDCs ranked by total admission frequency across all flow streams | | | | | |  |
